# Association between Diet Quality Index-International and the odds of polycystic ovary syndrome: a case–control study

**DOI:** 10.3389/fnut.2026.1986758

**Published:** 2026-10-30

**Authors:** Pushpamala Ramaiah, Ibtesam Nomani, Hayam Ibrahim Asfour, Gayrat Kodirov, Balakrishnan Rajalingam, Gulnora Yusupaliyeva, Shelly Agarwal, Safia Obaidur Rab, Bhawna Thukaral, Hamad AlMohamadi

**Affiliations:** 1 Faculty of Nursing, Umm Al-Qura University, Mecca, Saudi Arabia; 2 Jizzakh State Pedagogical University, Jizzakh, Uzbekistan; 3 Veltech Rangarajan Dr. Sagunthala R&D Institute of Science and Technology, Chennai, India; 4 Tashkent State Medical University, Tashkent, Uzbekistan; 5 Department of Obstetrics and Gynecology, Sharda University, Knowledge Park III, Greater Noida, India; 6 Department of Clinical Laboratory Sciences, College of Applied Medical Sciences, King Khalid University, Abha, Saudi Arabia; 7 Department of Nutrition and Dietetics, Chandigarh University, Mohali, Punjab, India; 8 Islamic University of Madinah, Madinah, Saudi Arabia

**Keywords:** diet, Diet Quality Index-International, DQI-I, PCOS, polycystic ovary syndrome

## Abstract

**Background:**

Polycystic ovary syndrome (PCOS) is the most common endocrine-metabolic disorder in women of reproductive age, associated with reproductive, metabolic, and psychological complications. Given the role of lifestyle factors in the pathophysiology of PCOS, dietary patterns have gained attention as modifiable determinants. However, previous studies have mostly focused on specific dietary indices, and the association between a comprehensive diet quality index and PCOS risk remains unclear. This study aimed to investigate the association between a comprehensive diet quality index and the odds of PCOS using a case–control design.

**Method:**

This case–control study enrolled 300 women (150 PCOS cases and 150 healthy controls) aged 20–35 years from Umm Al-Qura University Hospital. Dietary intake was assessed using a validated FFQ, and diet quality was measured by the Diet Quality Index-International (DQI-I) (score 0–100). Anthropometrics and physical activity were recorded. Logistic regression, adjusted for age, body mass index, smoking status, dietary fiber intake, physical activity, and total energy intake, was used to evaluate the association between DQI-I tertiles and PCOS risk, with *p* < 0.05 considered significant.

**Results:**

PCOS cases had higher BMI (29.1 vs. 27.0 kg/m^2^, *p* = 0.044) and lower DQI-I scores (68.0 vs. 70.0, *p* < 0.001) than controls, with lower intakes of fruits, vegetables, and low-fat dairy (*p* < 0.05) but higher MUFA and PUFA (*p* < 0.05). In adjusted logistic regression, the highest DQI-I tertile was associated with 70% lower odds of PCOS versus the lowest tertile (OR = 0.30, 95% CI: 0.16–0.56, P for trend <0.001), while the middle tertile was non-significant (OR = 0.78, 95% CI: 0.47–1.28).

**Discussion:**

Our findings demonstrated that overall diet quality, as measured by the DQI-I, was significantly lower among women with PCOS compared to healthy controls. This reduced diet quality remained independently and inversely associated with the odds of PCOS after adjustment for age, BMI, smoking, fiber, physical activity, and energy intake. Notably, women in the highest DQI-I tertile had approximately 70% lower odds of PCOS compared with those in the lowest tertile, an association that is both statistically robust and clinically meaningful.

## Introduction

Polycystic ovary syndrome (PCOS) is recognized as the most prevalent endocrine-metabolic disorder among women of reproductive age and is defined, according to the Rotterdam diagnostic criteria, by a combination of chronic oligo-ovulation or anovulation, clinical or biochemical hyperandrogenism, and polycystic ovarian morphology on ultrasonography (1). The prevalence of this syndrome varies considerably depending on the diagnostic criteria applied and the ethnic characteristics of the study population; global meta-analyses have estimated overall prevalence at approximately 9 to 12 percent, rising to more than 11 percent when the Rotterdam criteria are used (2, 3). Furthermore, studies based on the Global Burden of Disease framework have demonstrated a continuous upward trend over the past three decades in both prevalence and disability-adjusted life years (DALYs) attributable to PCOS, underscoring the substantial public health burden imposed by this disorder (3). The clinical heterogeneity of PCOS, spanning menstrual irregularities, infertility, hirsutism, and acne, is particularly notable among adolescents and young women (4).

From a pathophysiological standpoint, insulin resistance and compensatory hyperinsulinemia have been identified as the central axis underlying the onset and progression of PCOS. Hyperinsulinemia stimulates ovarian theca cells directly and suppresses hepatic synthesis of sex hormone-binding globulin (SHBG), thereby increasing circulating free androgen concentrations and exacerbating hyperandrogenism; conversely, excess androgens elevate free fatty acid levels and alter skeletal muscle composition, perpetuating the vicious cycle between insulin resistance and hyperandrogenism (5). This bidirectional interaction, together with chronic low-grade inflammation and oxidative stress, sets the stage for long-term metabolic complications, including type 2 diabetes, dyslipidemia, and cardiovascular disease. Moreover, this disorder is not confined to metabolic and reproductive dimensions alone but also exerts a significant negative impact on health-related quality of life; women with PCOS consistently report lower scores across physical, psychological, and social domains of quality of life compared with healthy counterparts (6).

Given the well-established role of environmental and lifestyle factors in modulating the pathophysiological pathways described above, dietary intake has emerged as one of the most important modifiable determinants in the prevention and management of PCOS. Notably, rather than examining single macronutrients or micronutrients in isolation, the diet quality index approach, which evaluates the overall pattern and simultaneous consumption of multiple food groups, has gained particular prominence owing to its more accurate reflection of real-world dietary behavior. Evidence from large cohort studies has shown that higher overall diet quality, as measured by indices such as the Healthy Eating Index or the Alternate Healthy Eating Index, is associated with reduced risk of all-cause, cardiovascular, and cancer mortality (7, 8). In the context of PCOS, several case–control studies have examined this association; for instance, a study conducted in Iran demonstrated that higher Healthy Eating Index scores were associated with lower odds of PCOS (9), whereas the Dietary Inflammatory Index was found to be directly and positively associated with increased odds of the syndrome (10). Similarly, the dietary phytochemical index (11), the Lifelines Diet Score (12), and food security-related dietary patterns (13) have each been reported to be associated with PCOS status across different populations. Consequently, adherence to a balanced dietary pattern rich in fruits, vegetables, whole grains, and unsaturated fatty acids appears capable of modulating the severity of PCOS manifestations through the attenuation of systemic inflammation and improvement of insulin sensitivity (14).

Despite this growing body of evidence, existing studies have predominantly focused on specific, narrowly defined indices such as the inflammatory index or the phytochemical index, and relatively few investigations have examined the association between a comprehensive diet quality index and the odds of PCOS within a case–control design. In addition, available findings lack sufficient consistency, a discrepancy that may be attributable to differences in study populations, dietary assessment instruments, and the methodology used to calculate diet quality indices (15, 16). Therefore, in light of this existing research gap and the clinical and public health significance of identifying modifiable nutritional determinants for the prevention of PCOS, the present study was designed and conducted to investigate the association between diet quality index and the odds of polycystic ovary syndrome using a case–control study design.

## Materials and methods

### Study population

This case–control investigation enrolled 300 women in total: 150 participants between 20 and 35 years of age who had either a history of or a confirmed diagnosis of polycystic ovary syndrome (PCOS), alongside 150 healthy, age-matched women serving as controls. All participants were drawn from endocrinology outpatient clinics affiliated with Umm Al-Qura University Hospital.

The sample size was calculated *a priori* using G*Power software (version 3.1.9.7). Assuming a two-sided α error of 0.05, a power of 80%, and an anticipated medium-to-large effect size (Cohen’s d = 0.5) for the primary comparison of metabolic and hormonal parameters between PCOS cases and controls, a minimum of 128 participants per group was required. To account for an anticipated 15% attrition or incomplete data, the target sample was increased to 150 women per group, yielding a total of 300 participants. This sample size also provided adequate power (>80%) for the secondary comparisons.

A total of 336 women were screened for eligibility. Of these, 36 women were excluded for the following reasons: 14 did not meet the Rotterdam criteria for PCOS, 8 were outside the 20–35-year age range, 7 reported a history of major chronic illness (cardiovascular disease, malignancy, diabetes, or renal disease), 5 had used medications capable of altering reproductive or metabolic parameters within the 3 months before enrollment (metformin or other insulin-sensitizing agents, GLP-1 receptor agonists, hormonal contraceptives, anti-androgen drugs, or any exogenous hormone therapy), and 2 had followed a specialized diet within the same period. After these exclusions, 300 women (150 PCOS cases and 150 controls) were enrolled and included in the analysis.

Women assigned to the PCOS group had to satisfy the Rotterdam consensus criteria (2003) for diagnosis, which require the presence of at least two of the following three features: irregular or absent ovulation, clinical and/or laboratory evidence of elevated androgens, and a polycystic ovarian appearance on ultrasound imaging. Eligibility was restricted to women whose diagnosis had been made within the preceding 3 months (17).

Controls were selected from women with no prior history or diagnosis of PCOS, drawn from a range of outpatient services including orthopedic, ENT, and elective pre-surgical clinics. Eligible controls needed to report regular cycles falling between 26 and 33 days, and each underwent a structured clinical interview and assessment by trained staff. To confirm the absence of hyperandrogenism or polycystic ovarian features, controls also had androgen panels drawn and pelvic ultrasounds performed. Apart from PCOS status, the same eligibility standards applied to both groups.

Women were excluded from participation if they reported a history of major chronic illness — cardiovascular disease, malignancy, diabetes, or renal disease. Also excluded were those who, in the 3 months before enrollment, had followed a specialized diet or had taken medications capable of altering reproductive or metabolic parameters, including metformin or other insulin-sensitizing agents, GLP-1 receptor agonists, hormonal contraceptives, anti-androgen drugs, or any exogenous hormone therapy.

### Dietary intake assessment

Habitual dietary intake over the past year was assessed using a semi-quantitative food frequency questionnaire (FFQ) specifically developed and validated for the Saudi population (18). The FFQ was originally designed by Aljohani and underwent rigorous validation procedures, including assessment of internal consistency (Cronbach’s α = 0.78), test–retest reliability (Pearson’s correlation coefficient = 0.78, *p* < 0.001), and criterion validity against 24-h dietary recalls. The validation study demonstrated that approximately 85% of food items reported in open-ended dietary recalls were captured by the FFQ, confirming its completeness for this population (18).

The questionnaire consisted of 152 food items organized into culturally appropriate categories. For each food item, participants reported their frequency of consumption using a 9-point categorical scale: never or less than once per month; 1–3 times per month; once per week; 2–4 times per week; 5–6 times per week; once per day; 2–3 times per day; 4–5 times per day; and 6 or more times per day. Participants selected their typical portion size from three to five predefined options for each food item.

Reported frequencies and portion sizes were converted to average daily nutrient and food-group intakes using the following procedure. First, the frequency response for each item was converted to a daily equivalent (e.g., “2–4 times per week” = 3/7 = 0.43 times per day). Second, this daily frequency was multiplied by the gram weight of the reported portion size to yield daily grams consumed for each food item. The FFQ was administered by trained interviewers in face-to-face sessions to minimize misinterpretation of portion sizes and food items.

### Diet quality assessment

Overall diet quality was evaluated using the Diet Quality Index-International (DQI-I), a comprehensive scoring system comprising four principal components. The first component, variety, reflects both the diversity of food groups consumed, including legumes, eggs, meat, vegetables, fruit, grains, and dairy, as well as the variety of protein sources derived from legumes, dairy, meat, and eggs, with a maximum score of 20. The second component, adequacy, captures the sufficiency of intake for essential nutrients, including protein, grains, fruit, vegetables, fiber, calcium, vitamin C, and iron, and is scored up to 40. The third component, moderation, assesses dietary restraint with respect to cholesterol, saturated fat, total fat, energy-dense and nutrient-poor foods, and sodium, contributing a maximum of 30 points. The fourth and final component, balance, evaluates the proportion of macronutrients and the ratios of fatty acids, with a possible score of 10. The summation of these four component scores yields a total DQI-I score ranging from 0, indicating the poorest diet quality, to 100, representing the best possible diet quality (19).

### Anthropometric and physical activity assessment

A trained dietitian carried out all anthropometric measurements. Weight was measured to the nearest 0.5 kg with participants in light clothing and without shoes, using a calibrated digital scale (Seca, Hamburg, Germany). Standing height was recorded to the nearest 0.5 cm with a non-stretch tape fixed to a flat wall, also without footwear. Body mass index was derived as weight in kilograms divided by the square of height in meters. Habitual physical activity levels were captured with a validated questionnaire and expressed as metabolic equivalent task-hours per day (MET-h/d) (20).

### Statistical analysis

All statistical analyses were performed using SPSS version 26.0 (IBM Corp., Chicago, IL, United States), and a two-sided *p*-value < 0.05 was considered statistically significant. The distribution of each continuous variable was assessed using the Kolmogorov–Smirnov or Shapiro–Wilk test, together with visual inspection of histograms and Q–Q plots. Variables that satisfied the assumption of normality were compared using the independent-samples *t*-test for two-group comparisons and one-way ANOVA for comparisons across DQI-I tertiles, whereas non-normally distributed variables were compared using the Mann–Whitney U test and the Kruskal–Wallis test, respectively. Group differences in baseline characteristics between cases and controls were tested using the chi-square test for categorical variables and either the independent-samples *t*-test or the Mann–Whitney U test for continuous variables, as appropriate. Categorical data are presented as percentages, while continuous data are reported as means ± standard deviation or medians with 25th–75th percentiles, depending on their distribution. Participants were further divided into tertiles according to their DQI-I scores, and differences in nutrient intake across these tertiles were examined using one-way ANOVA or the Kruskal–Wallis test, as appropriate. Multivariable logistic regression across DQI-I tertiles was used to estimate the association between diet quality and PCOS risk.

## Results

The baseline characteristics of the study participants are summarized in Table 1. The mean age of participants was 28.2 ± 6.3 years in the case group and 27.5 ± 5.8 years in the control group, with no statistically significant difference between the two groups (*p* = 0.118). However, body mass index (BMI) was significantly higher in cases compared to controls (median: 29.1 vs. 27.0 kg/m^2^, *p* = 0.044), while physical activity levels did not differ significantly between the groups (*p* = 0.091). Regarding dietary quality, the total DQI-I score was significantly lower in the case group than in the control group (median: 68.0 vs. 70.0, *p* < 0.001). Among the DQI-I subscales, the adequacy score and the moderation score were both significantly lower in cases compared to controls (*p* = 0.008 and *p* = 0.006, respectively), whereas no significant differences were observed for the variety score (*p* = 0.225) or the overall balance score (*p* = 0.660). Although energy and fiber intake were slightly higher in the case group, these differences did not reach statistical significance (*p* = 0.062 and *p* = 0.168, respectively).

**Table 1:** Baseline characteristics of the study population.

Variables	Cases (150)	Controls (150)	*P*-value
Age (year)	28.2 ± 6.3	27.5 ± 5.8	0.118
BMI (kg/m^2^)	29.1 (26.2–32.8)	27.0 (25.0–31.0)	0.044
Physical activity (MET-h/day)	31.8 (29.0–35.0)	33.2 (29.8–36.5)	0.091
Variety score	20.0 (20.0–20.0)	20.0 (20.0–20.0)	0.225
Adequacy score	37.5 (35.5–38.5)	38.5 (37.0–39.5)	0.008
Moderation score	6.5 (3.5–8.5)	7.0 (6.0–9.0)	0.006
Overall balance score	5.0 (4.0–6.0)	5.0 (4.0–6.0)	0.660
DQI-I total score	68.0 (65.0–70.0)	70.0 (67.0–74.0)	<0.001
Energy (kcal/day)	2650.8 (2200.5–2990.3)	2580.4 (2180.2–2850.7)	0.062
Fiber (g/day)	34.0 (27.0–43.5)	36.0 (27.5–49.0)	0.168
Smoking, no, %	140 (93.3)	145 (96.7)	0.621

BMI, body mass index; MET, metabolic equivalent of task; DQI-I, Dietary Quality Index-International. Values are presented as mean ± SD or median (25th–75th percentiles) for continuous variables and number (percent) for categorical variables.

^a^Independent samples *t*-test for normally distributed continuous variables.

^b^Mann–Whitney U test for non-normally distributed continuous variables.

^c^Chi-square test for categorical variables.

The consumption of food groups and macronutrients among cases and controls is presented in Table 2. Regarding macronutrient composition, no significant differences were observed between the two groups in terms of carbohydrate, protein, or saturated fatty acid (SFA) intake (*p* > 0.05 for all). However, the intake of monounsaturated fatty acids (MUFA) was significantly higher in the case group compared to the control group (13.0 ± 3.0 vs. 12.2 ± 2.8 energy %, *p* = 0.008), and a similar pattern was observed for polyunsaturated fatty acids (PUFA), with cases consuming significantly higher amounts than controls (8.3 ± 2.8 vs. 7.6 ± 2.9 energy %, *p* = 0.025). With respect to food groups, the case group had significantly lower consumption of fruits (median: 430.0 vs. 500.0 g/day, *p* = 0.017), vegetables (300.0 vs. 362.0 g/day, *p* = 0.029), and low-fat dairy products (565.0 vs. 670.0 g/day, *p* = 0.022) compared to the control group. No significant differences were observed between the two groups regarding the intake of whole grains, refined grains, red meats, poultry, fishes, processed meats, organ meats, high-fat dairy, legumes, nuts, snacks, sweets, liquid oils, solid oils, or sugar-sweetened beverages (*p* > 0.05 for all). These findings suggest that dietary patterns, particularly lower intakes of fruits, vegetables, and low-fat dairy products, along with higher intakes of unsaturated fatty acids, may be associated with the disease status in the study population.

**Table 2:** Food group consumption based on the case and control groups.

Variables	Cases (*n* = 150)	Controls (*n* = 150)	*P*-value
Carbohydrate (energy %)	54.5 ± 6.9	54.8 ± 7.2	0.518
Protein (energy %)	12.5 ± 2.2	12.8 ± 2.0	0.108
SFA (energy %)	11.0 ± 2.1	10.6 ± 2.4	0.152
MUFA (energy %)	13.0 ± 3.0	12.2 ± 2.8	**0.008**
PUFA (energy %)	8.3 ± 2.8	7.6 ± 2.9	**0.025**
Whole grains (g/day)	72.5 (35.0–113.0)	68.0 (40.0–118.0)	0.345
Refined grains (g/day)	278.5 (206.0–338.0)	285.0 (182.0–390.0)	0.392
Fruits (g/day)	430.0 (336.0–585.0)	500.0 (365.0–648.0)	**0.017**
Vegetables (g/day)	300.0 (222.0–475.0)	362.0 (270.0–465.0)	**0.029**
Red Meats (g/day)	19.0 (10.0–29.0)	19.5 (13.0–29.0)	0.468
Poultry (g/day)	55.0 (27.0–74.0)	55.0 (28.0–81.0)	0.702
Fishes (g/day)	7.5 (3.5–16.0)	9.5 (4.0–18.5)	0.132
Processed meats (g/day)	1.5 (0.2–6.0)	1.2 (0.0–4.5)	0.178
Organ meats (g/day)	3.5 (0.7–7.5)	2.5 (0.8–6.5)	0.112
Low-fat dairy (g/day)	565.0 (320.0–812.0)	670.0 (425.0–910.0)	**0.022**
High-fat dairy (g/day)	42.0 (13.0–234.0)	48.0 (13.0–238.0)	0.876
Legumes (g/day)	23.5 (14.0–39.0)	27.5 (15.5–44.5)	0.248
Nuts (g/day)	8.0 (3.0–15.5)	9.5 (4.0–16.5)	0.055
Snacks (g/day)	16.5 (8.0–38.5)	16.5 (5.5–44.5)	0.588
Sweets (g/day)	44.0 (26.0–71.0)	38.0 (24.0–57.0)	0.095
Liquid oils (g/day)	17.5 (9.0–24.5)	15.5 (6.5–26.0)	0.718
Solid oils (g/day)	15.0 (5.5–27.0)	11.0 (3.5–27.0)	0.082
Sugar-sweetened beverages (g/day)	16.5 (2.0–41.0)	8.5 (1.5–35.5)	0.075

DQI-I, Dietary Quality Index-International; SFA, saturated fatty acids; MUFA, monounsaturated fatty acids; PUFA, polyunsaturated fatty acids; g, gram. Values are presented as mean ± SD or median (25th–75th percentiles) for continuous variables. Using independent samples *t*-test for normally distributed continuous variables. Using Mann–Whitney U test for non-normally distributed continuous variables. Bold values indicate statistically significant results.

The association between DQI-I tertiles and PCOS risk is presented in Table 3. In the model adjusted for age (Model 1), participants in the highest tertile of DQI-I (T3, ≥70.51) had significantly lower odds of PCOS compared to those in the lowest tertile (T1, ≤66.00), with an odds ratio of 0.28 (95% CI: 0.15–0.51). No significant association was observed for the intermediate tertile (T2, 66.01–70.50) compared to the reference group (OR = 0.77, 95% CI: 0.47–1.26). After adjustment for multiple confounding variables, including age, body mass index, smoking, fiber, physical activity, and energy intake (Model 2), the results remained essentially unchanged, with the highest tertile continuing to show a significantly reduced odds of PCOS (OR = 0.30, 95% CI: 0.16–0.56) while the middle tertile remained non-significant (OR = 0.78, 95% CI: 0.47–1.28). Furthermore, a significant trend was observed across increasing tertiles of DQI-I in both models (P for trend < 0.001), indicating that higher diet quality, as reflected by higher DQI-I scores, is associated with a lower likelihood of PCOS in a graded manner.

**Table 3:** Odds ratios and 95% confidence intervals for the association between Diet Quality Index-International (DQI-I) tertiles and PCOS risk.

Tertiles of DQI-I	Cases (*n*)	Controls (*n*)	Unadjusted OR (95% CI)	Model 1 OR (95% CI)	Model 2 OR (95% CI)	Model 2a OR (95% CI)	Model 2b OR (95% CI)
T1 (≤66.00)	65	35	1.00 (Ref.)	1.00 (Ref.)	1.00 (Ref.)	1.00 (Ref.)	1.00 (Ref.)
T2 (66.01–70.50)	50	50	0.78 (0.48–1.27)	0.77 (0.47–1.26)	0.78 (0.47–1.28)	0.77 (0.47–1.27)	0.76 (0.46–1.25)
T3 (≥70.51)	35	65	0.29 (0.16–0.52)	0.28 (0.15–0.51)	0.30 (0.16–0.56)	0.31 (0.16–0.58)	0.32 (0.17–0.60)
P for trend	–	–	<0.001	<0.001	<0.001	<0.001	<0.001

DQI-I, dietary quality index-international; PCOS, polycystic ovary syndrome; OR, odds ratio; CI, confidence interval.

Model 1: adjusted for age.

Model 2: adjusted for age, body mass index, smoking, fiber, physical activity, and energy intake.

Model 2a: sensitivity analysis—Model 2 without adjustment for fiber.

Model 2b: sensitivity analysis—Model 2 without adjustment for body mass index.

P for trend was calculated by assigning the median value of each tertile as a continuous variable in the logistic regression models.

Values are presented as odds ratio (95% confidence intervals). Significant values are shown in bold.

Covariates included in Model 2 were selected a priori based on established biological relevance to both dietary intake and PCOS risk (age, BMI) and were supplemented by variables that were either associated with case–control status at *p* < 0.20 in bivariate analysis or recognized in the literature as potential confounders of the diet–PCOS association (smoking status, physical activity, dietary fiber intake, and total energy intake). Bold values indicate statistically significant results.

## Discussion

The present case–control study demonstrated that overall diet quality, as measured by the Diet Quality Index-International (DQI-I), was significantly lower among women with PCOS compared with age-matched healthy controls, and that this reduced diet quality was independently and inversely associated with the odds of PCOS, even after adjustment for age, BMI, smoking, fiber, physical activity, and energy intake. Women in the highest DQI-I tertile had approximately 70% lower odds of PCOS relative to those in the lowest tertile, a magnitude of association that is both statistically robust and clinically meaningful.

These findings are consistent with, and extend, a growing body of literature linking overall dietary quality, rather than single nutrients or foods in isolation, to PCOS risk. Prior Iranian case–control studies have reported that higher Healthy Eating Index scores are associated with lower odds of PCOS (9), whereas a pro-inflammatory dietary pattern, as captured by the Dietary Inflammatory Index, increases PCOS odds (10). Similarly, higher adherence to the dietary phytochemical index (11) and to the Lifelines Diet Score (12) has been associated with reduced PCOS odds, while adverse food-security status and a quasi-Western dietary pattern have been linked to higher odds of the syndrome (13). Our results align with this pattern and reinforce it using a more comprehensive, internationally validated index that simultaneously captures variety, adequacy, moderation, and balance. Comparable inverse associations between overall diet quality and PCOS-related outcomes have also been reported using other composite indices, including the Prime Diet Quality Score combined with Mediterranean diet adherence (21), empirically derived “mixed” dietary patterns (22), and Mediterranean-style dietary patterns in large community cohorts (23). By contrast, Western and plant-based factor-derived patterns, driven largely by refined grains, red meat, and sugar-sweetened items, have been shown to increase PCOS odds (13), a pattern broadly compatible with the lower adequacy and moderation subscale scores observed among our cases. Notably, similar dietary shortfalls have been reported within the Saudi population itself: a comparative study of food intake in Saudi women with PCOS found meaningful differences in habitual intake relative to controls (24), and fast-food and junk-food consumption has been specifically associated with PCOS and its clinical features (facial acne, weight gain) among reproductive-aged women in Jeddah (25), lending regional plausibility to our findings.

Examination of the DQI-I subcomponents provides further mechanistic texture to this overall association. The adequacy score, which reflects sufficiency of intake of protein, grains, fruit, vegetables, fiber, calcium, vitamin C, and iron, and the moderation score, which reflects dietary restraint regarding saturated fat, cholesterol, energy-dense foods, and sodium, were both significantly lower among cases, whereas variety and overall balance scores did not differ. This pattern was corroborated at the food-group level, where cases consumed significantly less fruit, vegetables, and low-fat dairy than controls. Fruit and vegetable intake supplies fiber, potassium, and antioxidant phytochemicals that have been linked to improved insulin sensitivity and attenuation of low-grade inflammation in PCOS (11), while low intakes of dietary fiber have separately been associated with insulin resistance and hyperandrogenism in women with PCOS (26). Reduced consumption of low-fat dairy is more difficult to interpret mechanistically, since meta-analytic evidence on dairy intake and PCOS-related metabolic and androgenic outcomes remains inconsistent, with clinical trials suggesting that dairy reduction (rather than low intake per se) can improve insulin and androgen profiles (27); it is therefore plausible that the specific type and fat content of dairy consumed, rather than dairy intake as a category, is the more relevant exposure, a nuance that FFQ-based food-group analyses cannot fully resolve. A lower adequacy score also implies lower intake of micronutrients such as calcium and, indirectly, vitamin D, which is frequently reported as insufficient in women with PCOS and has been associated with metabolic disturbances in this population (28); although randomized trials of vitamin D supplementation on insulin sensitivity in PCOS have produced mixed results (29), the coexistence of low adequacy scores and known micronutrient shortfalls in PCOS supports the biological coherence of our findings.

An apparently counterintuitive finding was the significantly higher intake of monounsaturated (MUFA) and polyunsaturated (PUFA) fatty acids among cases compared with controls. This contrasts with several prior reports in which PCOS was associated with lower MUFA and n-3 PUFA intake relative to controls (30) and with evidence that dietary long-chain omega-3 PUFA intake is inversely associated with insulin resistance and adiposity in PCOS (31). Experimental data further indicate that the metabolic consequences of increasing PUFA intake in PCOS are not uniformly favorable: a controlled feeding study substituting PUFA for saturated fat improved free fatty acid and ketone profiles but paradoxically worsened fasting glucose and glucose tolerance, without measurable change in insulin, androgens, or SHBG (32). Taken together with our results, this suggests that the quantity of unsaturated fat captured by a food-composition database may not adequately reflect its quality or dietary source (for example, refined vegetable and seed oils and processed snack foods versus olive oil, nuts, and fatty fish), and that the n-6 to n-3 ratio, rather than total PUFA intake, may be the more biologically relevant exposure. Because the DQI-I balance component scores macronutrient and fatty-acid ratios rather than absolute fat quality, this dimension of dietary exposure may be incompletely captured by the index, representing a plausible explanation for this discordant subfinding and an area warranting more granular dietary assessment in future PCOS nutrition research. It should be emphasized that the biological explanations proposed above are hypotheses derived from our observations and the existing literature; the present study did not directly measure fatty-acid source, quality, or the n-6:n-3 ratio, and these mechanisms therefore remain to be tested directly in future research.

Beyond direct nutrient effects, several converging biological pathways can plausibly account for the observed association between poor diet quality and PCOS odds. Diets low in fiber-rich fruits, vegetables, and whole grains provide fewer substrates for colonic fermentation to short-chain fatty acids, and PCOS has been consistently associated with gut microbial dysbiosis and reduced short-chain-fatty-acid-producing taxa (33), a pattern also documented in obese adolescents with PCOS (34). Because short-chain fatty acids modulate insulin sensitivity, gut barrier integrity, and systemic inflammation, diet-driven dysbiosis represents a mechanistically coherent intermediate step linking a low-adequacy, low-moderation dietary pattern to the insulin resistance and compensatory hyperinsulinemia that underlie hyperandrogenism and anovulation in PCOS (5). This is further supported by the observation that a low-inflammatory, nutrient-adequate Mediterranean-style diet is independently associated with more favorable insulin resistance and hyperandrogenemia profiles in PCOS (30), and that an 8-week DASH dietary intervention, rich in fruit, vegetables, whole grains, and low-fat dairy, significantly improved insulin resistance and hs-CRP in women with PCOS in a randomized controlled trial (35), providing experimental, causally informative support for the associations we observed cross-sectionally.

It is notable that no significant between-group difference was found for total carbohydrate intake, despite carbohydrate quality being repeatedly implicated in PCOS pathophysiology. A systematic review and meta-analysis of randomized controlled trials has shown that lowering dietary glycemic index and glycemic load, independent of total carbohydrate quantity, improves insulin sensitivity and reproductive parameters in PCOS (36). Because the DQI-I does not explicitly score glycemic index or glycemic load, our finding of no difference in carbohydrate quantity alongside a significant difference in overall diet quality is not contradictory but rather suggests that carbohydrate quality, a dimension only partially captured through the adequacy and moderation subscales, may be an important additional axis of dietary risk in this population that merits dedicated assessment in future studies.

The robustness of the association after adjustment for BMI is noteworthy, given that BMI itself was significantly higher among cases. This finding suggests that the association between diet quality and PCOS odds may operate, through pathways independent of adiposity, consistent with evidence from other DQI-I-based case–control and cohort studies showing independent inverse associations between DQI-I and other insulin-resistance-related conditions, including non-alcoholic fatty liver disease (37) and type 2 diabetes (38), as well as favorable associations with metabolic risk factors in a large Middle Eastern cohort (39). The consistency of the DQI-I’s performance across these metabolically related conditions strengthens confidence in the biological plausibility and external validity of the present findings and supports the use of this index as a generalizable tool for capturing diet-related endocrine-metabolic risk. Restricting PCOS-group eligibility to women diagnosed within the preceding 3 months also reduces, though does not eliminate, the possibility that post-diagnosis dietary counseling or symptom-driven dietary modification accounts for the observed differences, an important methodological strength relative to studies enrolling women with long-standing disease.

From a clinical standpoint, our findings are consistent with current international recommendations that place lifestyle and dietary modification at the center of first-line PCOS management (40). Given that overall diet quality, rather than any single nutrient, appears to distinguish women with PCOS from healthy controls in the present sample, dietary counseling programs for PCOS may benefit from targeting the DQI-I’s adequacy and moderation domains specifically (increasing fruit, vegetable, whole-grain, and low-fat dairy intake while limiting energy-dense, nutrient-poor foods) rather than focusing narrowly on macronutrient ratios alone.

This study has several limitations that warrant consideration. First, the case–control design precludes causal inference and cannot establish temporality between poor diet quality and PCOS onset; reverse causation, whereby early metabolic or psychological consequences of PCOS alter eating behavior, cannot be entirely excluded despite the recent-diagnosis restriction. Second, dietary intake was self-reported via an FFQ, which is subject to recall bias, social desirability bias, and measurement error, particularly among women with higher BMI, and any resulting non-differential misclassification would likely have biased associations toward the null. Third, participants were recruited from a single tertiary hospital’s outpatient clinics, which may limit generalizability to community-based or other ethnic populations, although the directionally consistent findings reported in other Saudi cohorts (24, 25) offer some reassurance regarding regional applicability. Fourth, PCOS is a clinically heterogeneous syndrome, and phenotype-specific analyses (for example, hyperandrogenic versus normoandrogenic phenotypes) were not performed, potentially obscuring differential diet-phenotype relationships. In addition, hospital-based recruitment of both cases and controls may have introduced selection bias, potentially limiting generalizability to community-dwelling women. Residual confounding by unmeasured or imprecisely measured factors, such as socioeconomic status and detailed physical activity patterns, cannot be excluded. Furthermore, because BMI and physical activity may lie partly on the causal pathway linking diet quality to PCOS rather than acting solely as confounders, the possibility of overadjustment in the multivariable model should be considered when interpreting the adjusted odds ratios. Finally, although eligibility was restricted to women diagnosed within the preceding 3 months to minimize disease-related dietary modification, some degree of post-diagnosis dietary change cannot be entirely ruled out. Finally, biochemical correlates of insulin resistance, hyperandrogenism, and vitamin D status (for example, HOMA-IR, free androgen index, SHBG, 25-hydroxyvitamin D) were not measured in relation to DQI-I scores, limiting our ability to directly test the mechanistic pathways proposed above.

## Conclusion

In conclusion, our findings suggest that a comprehensive, higher-quality dietary pattern, characterized by adequate intake of fruits, vegetables, and low-fat dairy and moderation of energy-dense, nutrient-poor foods, is independently associated with lower odds of PCOS in a graded manner, while highlighting an unexpected pattern of higher unsaturated fat intake among cases that likely reflects fat quality or source rather than quantity. These findings support consideration of comprehensive diet-quality assessment and counseling as part of PCOS management strategies and, pending confirmation in prospective studies, as a component of risk-reduction approaches.

## Data Availability

The raw data supporting the conclusions of this article will be made available by the authors, without undue reservation.
